# Measuring molecular frequencies in the 1–10 μm range at 11-digits accuracy

**DOI:** 10.1038/s41598-017-12891-6

**Published:** 2017-10-06

**Authors:** G. Insero, S. Borri, D. Calonico, P. Cancio Pastor, C. Clivati, D. D’Ambrosio, P. De Natale, M. Inguscio, F. Levi, G. Santambrogio

**Affiliations:** 1 Istituto Nazionale di Ottica-CNR & Dipartimento di Fisica e Astronomia, Università di Firenze & European Laboratory for Non-Linear Spectroscopy LENS, Via Nello Carrara 1, 50019 Sesto Fiorentino, Italy; 2 INFN, Istituto Nazionale di Fisica Nucleare, Sez. di Firenze, Via Nello Carrara 1, 50019 Sesto Fiorentino, Italy; 3 Istituto Nazionale di Ricerca Metrologica INRIM, Strada delle Cacce 91, 10135 Torino, Italy

## Abstract

High-resolution spectroscopy in the 1–10 μm region has never been fully tackled for the lack of widely-tunable and practical light sources. Indeed, all solutions proposed thus far suffer from at least one of three issues: they are feasible only in a narrow spectral range; the power available for spectroscopy is limited; the frequency accuracy is poor. Here, we present a setup for high-resolution spectroscopy, whose approach can be applied in the whole 1–10 μm range. It combines the power of quantum cascade lasers (QCLs) and the accuracy achievable by difference frequency generation using an orientation patterned GaP crystal. The frequency is measured against a primary frequency standard using the Italian metrological fibre link network. We demonstrate the performance of the setup by measuring a vibrational transition in a highly-excited metastable state of CO around 6 μm with 11 digits of precision.

## Introduction

The fractional accuracy on spectroscopic measurements on atoms has reached the few parts in 10^18^ ^1^; the same quantity in experiments on molecules is worse by more than three orders of magnitude^2–7^. This difference is due to the richer internal structure of molecules that makes cooling and detection more complicated than in atoms. Nevertheless, the internal structure and symmetry of molecules, and their strong intramolecular fields can enable totally new measurements. Recent experiments on ThO yielded the most sensitive measurement to date of the electron electric dipole moment^2^. The upper limit found in the 10^−29^ *e* cm range constrains T-violating physics at the TeV energy scale, comparable to the energy scales explored directly at the Large Hadron Collider. In other experiments, the assessments in the laboratory of the variation of fundamental constants based on molecular spectroscopy^3,4^ achieve a level of sensitivity similar to astronomical observations looking back in time several billion years. Still other experiments are probing energy differences in enantiomers of chiral species^5^, testing quantum electrodynamics^6^, and searching for a fifth force^7^.

The mid IR is a natural spectral region for high-resolution spectroscopic studies on molecules because it corresponds to the typical energy range of fundamental rovibrational transitions, characterized by strong linestrenghts and Hz-level natural linewidths. In order to achieve a level of precision typical of atomic physics experiments, one needs a cold molecular sample combined with state-of-the-art light sources in the mid IR^8^. Since 2014, cold molecules technology has considerably advanced. YO^9^ and CaF^10^ were laser cooled and SrF was trapped in a three-dimensional magneto-optical trap^11^. Prehn *et al*.^12^ applied optoelectrical cooling to formaldehyde, achieving temperatures as low as 0.5 mK with 10^7−8^ molecules/cm^3^ densities. Very recently, Cheng *et al*.^13^ reported on an ammonia fountain that enables, in principle, measurements with sub-Hz linewidths; and Truppe *et al*.^14^ laser cooled CaF below the Doppler limit, to 50 μK.

However, photonics in the mid IR remains challenging. Coherent sources for precision spectroscopy must feature sufficient intensity, very narrow linewidth, high frequency stability, and an absolute frequency traceability against the primary frequency standard. Continuous wave optical parametric oscillators can match these requirements only below 5 μm but are difficult to operate^15^, while difference frequency generation processes suffer from low emitted powers. Room-temperature quantum cascade lasers (QCLs) are a promising technology. They cover the whole 3–25 μm range and yield mW-to-W power levels^16^. They have very narrow intrinsic linewidths^17^ but are particularly sensitive to driver current noise^18^. However, with proper control, QCLs have yielded linewidths in the sub-Hz–kHz range^19–21^. An open issue in using QCLs to perform precision spectroscopy is that most of a QCL emitted power has to be used to realize an up-frequency conversion in order to compare the QCL frequency to the frequency standard^20–22^.

This transfer of the frequency standard to the mid IR is generally achieved in two steps. First, an optical frequency comb bridges the gap from the microwave to the near IR. Then, a non-linear frequency conversion process is used to reach the mid IR. The accuracy of the IR frequencies depends critically on the stability of the comb repetition rate. The recent rise of frequency dissemination by optical fibres represents a radical improvement^23^. While RF oscillators disciplined by the Global Positioning System (GPS) can achieve 10^−14^ stability and accuracy only after tens of thousands seconds integration times, the same stability and accuracy are reached in 1 second by using an optical dissemination of the frequency standard, and the intrinsic uncertainty of the disseminated clock is reached with short interaction times^3,21,23^.

Here we demonstrate a laser technology that can be extended to the whole mid IR range between 1 and 10 μm with state-of-the-art metrological features. We demonstrate its performance by measuring a vibrational transition around 6 μm on a highly-excited, metastable state in carbon monoxide with kHz-level accuracy. The measurement is performed on a molecular beam with a density of about 10^8^/cm^3^ in a selected quantum state, similar to what is currently obtained in state-of-the-art setups for cold molecules. Our system is based on three pillars.

*First*, the frequency reference to the primary standard is transferred by an ultra stable laser at 1542 nm sent over the Italian fibre-link network^24^. This network connects several laboratories all over the Italian territory, and it is part of a continental metrological network that is currently under construction.

*Second*, difference frequency generation in an orientation patterned (OP) GaP crystal allows us to bridge the gap between near and mid IR. OP-GaP provides a reasonable efficiency^25^ and a high transparency over the whole 1–10 μm range, with an absorption coefficient always below 0.5 cm^−1^ ^26^. Unlike competing crystals like OP-GaAs, its band edge lies in the visible region, so two-photon absorption is negligible above 1 μm (for comparison, two-photon absorption in OP-GaAs prevents pumping below 1.5 μm)^27^. This allows OP-GaP to be pumped directly with powerful lasers at 1 μm, and proper domain patterning period provides the required tuning parameter needed to cover the material’s full transparency range.

*Third*, a quantum cascade laser produces the mid IR radiation, which is almost entirely used for spectroscopy and not for frequency control.

## Optical Setup

**Figure 1:**
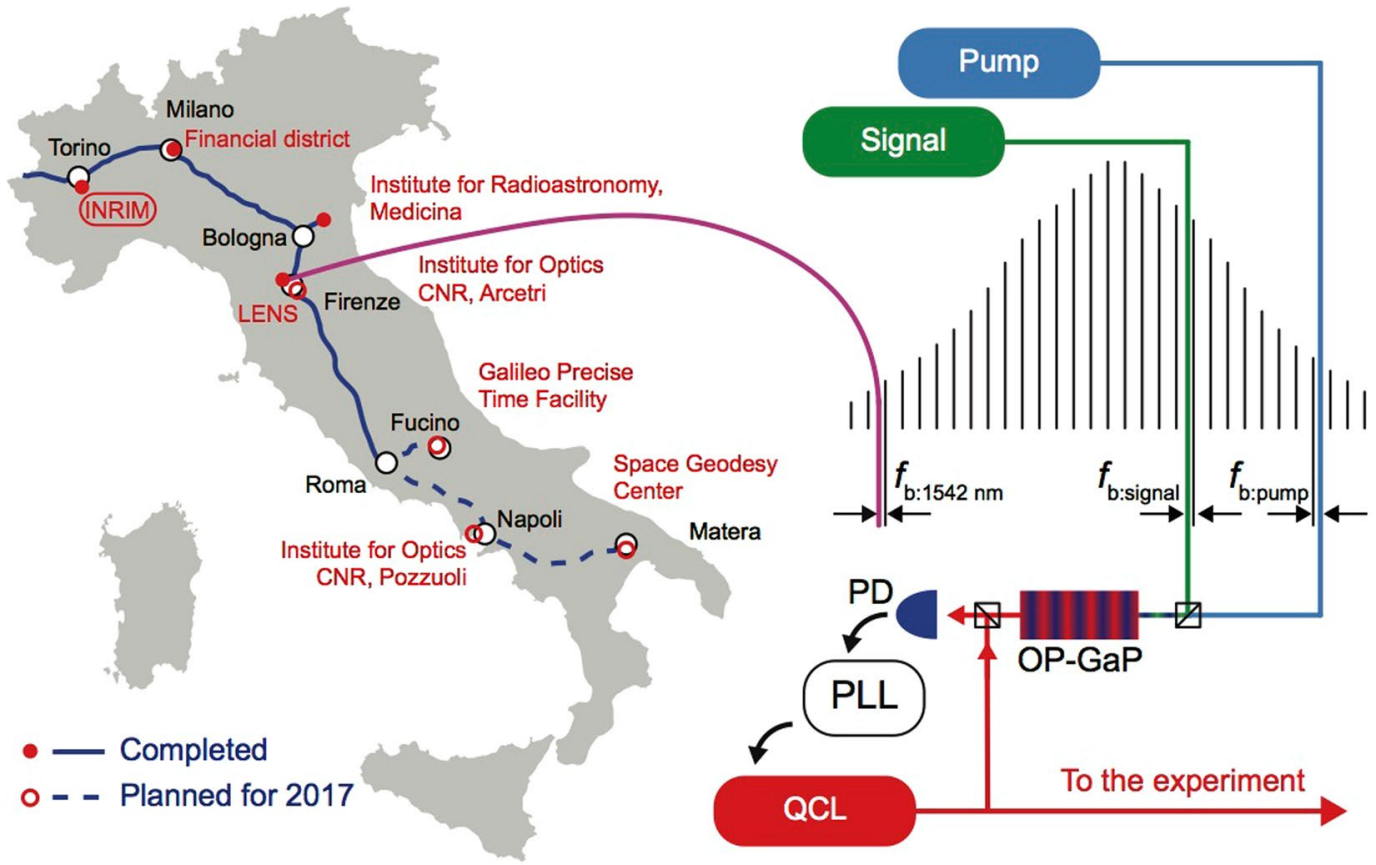
The left part of the figure shows the Italian fibre link network, blue line, with network nodes in red. At LENS/University of Florence (LENS in the following for brevity), the ultra stable laser at 1542 nm locks the repetition rate of a frequency comb. We measure the beat notes of two near-IR lasers against the frequency comb and keep their frequency difference constant with an indirect locking scheme to cancel out the comb noise contribution^29^. The two lasers are combined in an OP-GaP crystal to generate mid-IR light, to which we lock a quantum cascade laser. (The map is obtained on the basis of ©OpenStreetMap contributors (www.openstreetmap.org) licensed under the Creative Commons Attribution-ShareAlike 2.0 license (CC BY-SA) https://creativecommons.org/licenses/by-sa/2.0/. The basis was then modified to fit our figure and we took no care to maintain proportions or accuracy).

Figure 1 shows the main features of the metrological chain to control a QCL. We lock two near-IR lasers to a frequency comb whose repetition rate is referenced and stabilized using the fibre link. The light of these two lasers is mixed in an OP-GaP crystal to generate the difference of their frequency, which lies in the mid IR. Finally, such mid IR radiation is used to phase-lock the QCL.

The fibre link network carries the light of an ultra stable laser that is kept at a frequency of 194399996000000.00(4) Hz (i.e. around 1542 nm), where the uncertainty is directly related to that of the Cs fountain. The laser frequency is referenced to the Italian timescale, generated at the Italian Metrological Institute (INRIM) in Turin. This signal reaches several relevant locations on the national territory: the Institute for Radioastronomy in Medicina (535 km from INRIM), the European Laboratory for Nonlinear Spectroscopy, LENS, in Sesto Fiorentino (642 km), Rome (994 km), and the Italian-French border (150 km). In addition, a timestamp signal referenced to the Italian timescale reaches the financial district in Milan (279 km). During 2017, the fibre network will be extended to the National Institute for Optics INO in Arcetri (662 km), to CNR labs in Pozzuoli (1306 km), to the Galileo Precise Time Facility in Fucino (1134 km), to the Space Geodesy Center in Matera (1684 km), and finally to the French metrological institute LNE-SYRTE in Paris.

A series of bidirectional erbium-doped fibre amplifiers compensate for the almost 200-dB optical losses of the link between INRIM and LENS^24^. To phase-stabilize the fibre link, part of the radiation at 1542 nm travels back to INRIM on the same fibre, after being retro-reflected at LENS. The photon round-trip, over 1200 km, limits the bandwidth of the phase-lock loop. Thus the laser at 1542 nm has a large phase noise in the short term (<5 ms), while it has a stability of 1 × 10^−14^ at 1 s and an accuracy of 2 × 10^−16^; these performances have been assessed by measuring the absolute frequency of the 1542-nm laser on two independent optical combs referenced to the same H-maser^23^. The noise generated by the optical link does not affect the uncertainty of the delivered signal within parts in 10^19^ ^24^.

At LENS, we phase-lock a diode laser to the incoming radiation, replicating the stability of the link laser and boosting the optical power at a suitable level for referencing a frequency comb. An intra-cavity electro-optic modulator phase-locks the repetition rate *f*_rep_ of a commercial frequency comb to the 1542-nm light, with a bandwidth of approximately 300 kHz. We choose the lock frequency so that the repetition rate is 100 MHz when the incoming radiation is at the nominal frequency value. The carrier-envelope offset frequency *f*_0_ of the comb is stabilized to an RF frequency standard. The residual noise of the stabilized link, which scales with the link length^28^, dominates the phase-noise of the comb.

**Figure 2:**
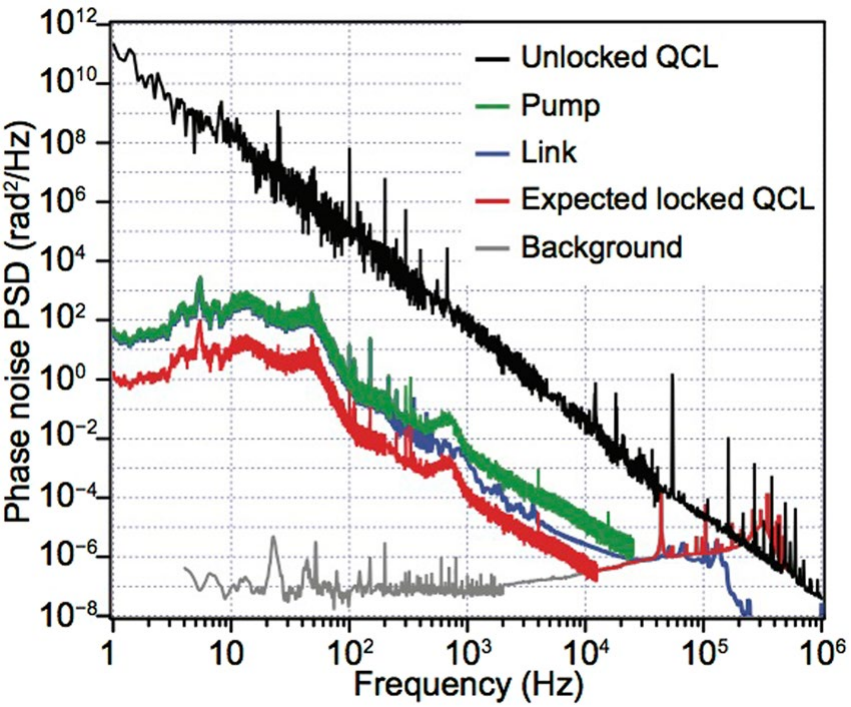
Phase noise of various components of the frequency locking chain. The phase noise of the free running QCL is shown in black. The noise on the fibre link is shown in blue. The error signal of the phase-lock loop for the pump laser is shown in green. The expected phase noise of the phase-locked QCL is shown in red and is obtained by scaling the phase noise of the pump laser, following the virtual beat note scheme.

In an OP-GaP crystal we generate the difference frequency of two mid IR lasers, a Nd:YAG MOPA system at 1064 nm with a linewidth of 1 kHz and operated at a power of about 5 W (pump), and a diode laser at 1220–1320 nm delivering about 30 mW (signal). The quasi phase matching condition is tuned between 5.8 and 6.0 μm (idler wavelength) by changing the temperature of the OP-GaP crystal between 30 and 150 °C. Two fast InGaAs photodiodes detect the beat notes of both lasers to the closest teeth of the frequency comb with a minimum signal-to-noise ratio of 25 dB on a 100 kHz bandwidth. We refer to these beat notes as ${f}_{{\rm{b}}:{\rm{pump}}}$ and ${f}_{{\rm{b}}:{\rm{signal}}}$, and we refer to the absolute frequencies of the two lasers as ${\nu }_{{\rm{pump}}}$ and ${\nu }_{{\rm{signal}}}$. The pump laser is phase-locked directly to the closest comb tooth on a bandwidth of 700 Hz. Figure 2 shows the phase noise of the beat note ${f}_{{\rm{b}}:{\rm{pump}}}$. Instead of locking the signal laser directly to the comb, we stabilize the difference frequency ${\nu }_{{\rm{pump}}}-{\nu }_{{\rm{signal}}}$ with an indirect lock of the signal laser to the pump. For this, we use the frequency comb to bridge the gap between the two lasers but we reject its noise contribution by proper processing of ${f}_{{\rm{b}}:{\rm{pump}}}$, ${f}_{{\rm{b}}:{\rm{signal}}}$, and *f*_0_^29^.

To implement this indirect locking scheme, we subtract *f*_0_ from each beat note using analog mixers, obtaining ${\overline{f}}_{{\rm{b}}:{\rm{pump}}}$ and ${\overline{f}}_{{\rm{b}}:{\rm{signal}}}$. Thus, the absolute frequencies of the two lasers can be written as ${\nu }_{{\rm{pump}}}={N}_{{\rm{pump}}}{f}_{{\rm{rep}}}+{\overline{f}}_{{\rm{b}}:{\rm{pump}}}$ and ${\nu }_{{\rm{signal}}}={N}_{{\rm{signal}}}{f}_{{\rm{rep}}}+{\overline{f}}_{{\rm{b}}:{\rm{signal}}}$, where *N*_pump_ = 2816363 and *N*_signal_ = 2302371 are the comb teeth used for the beat notes. A 14-bit direct digital synthesizer and a mixer generate the signal ${\overline{f}}_{{\rm{b}}:{\rm{signal}}}-({N}_{{\rm{signal}}}/{N}_{{\rm{pump}}}){\overline{f}}_{{\rm{b}}:{\rm{pump}}}$. This signal is phase-locked to a radio frequency reference and it feeds back the signal laser on a bandwidth exceeding 200 kHz. The finite number of digits of the digital synthesizer generates a bias of about 1 Hz in the frequency difference. However, this is calculated and corrected in the final results.

Finally, we phase lock the QCL to the difference frequency of the two mid IR lasers. 100 μW radiation from the QCL is enough to produce a beat note with good signal-to-noise ratio, suited for a robust phase lock. Therefore, almost all the QCL output power is available for spectroscopy. This is a big advantage with respect to those methods that reference the QCL frequency by up-conversion, which typically require over 10 mW of QCL power^20,21,30^.

We analyzed the phase noise power spectral density (PSD) of every step of the chain to characterize the metrological properties of the mid IR light. The link noise (shown in blue in Fig. 2) is derived from independent measurements^23^ and is re-scaled to the relevant spectral region. We estimate the residual noise of the pump laser at closed phase-lock-loop (PLL) (green trace in Fig. 2) to be limited by the comb noise within the PLL bandwidth of 700 Hz. We expect the phase noise PSD of the mid IR radiation produced in the OP-GaP crystal to follow that of the pump laser scaled by a factor ${\mathrm{(1}-{N}_{s}/{N}_{p})}^{2}$, which corresponds to a 15-dB noise reduction, because the noise introduced by the PPL of the indirect locking scheme is negligible. We phase lock the QCL to this mid IR radiation with a 300-kHz bandwidth PLL. The noise of this PLL is shown in gray in Fig. 2. When the phase-lock chain is operating, the phase noise of the QCL is reduced by 11 decades at 1 Hz, 7 decades at 10 Hz, and by over four decades at 10 kHz (black and red traces for free-running and locked, respectively). Hence, we infer that the QCL linewidth is limited by the Nd:YAG laser and that the QCL long term stability and accuracy reflects reliably the performance of the Cs fountain. Finally, we remark that QCL linewidth and jitter at the kHz level can be further improved by referencing the frequency comb to an ultra narrow optical-link-disciplined laser around the Nd:YAG frequency.

## Measurement of Molecular Frequencies

We prove the versatility of our setup by measuring a vibrational transition on a slow (318 m/s) molecular beam of highly-excited, metastable CO molecules, in a triple resonance experiment. Most of the QCL power can be coupled to the molecular beam, i.e. about 10 mW. For a typical transition dipole moment of 0.05 debye, this power corresponds to a Rabi frequency of some MHz for a beam waist of 1 mm, which are comfortable numbers for many experiments.

**Figure 3:**
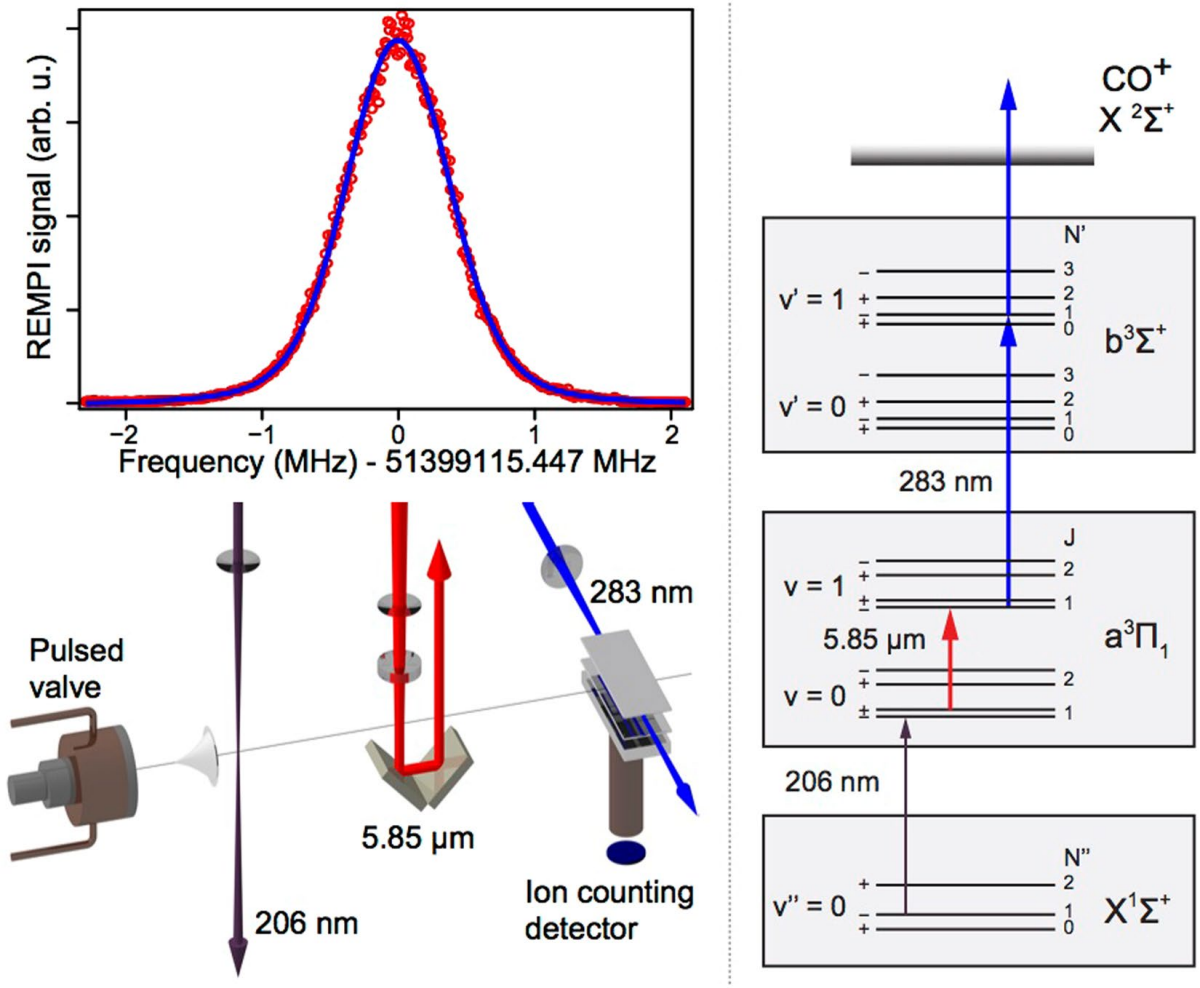
Top: a typical vibrational absorption spectrum on the ${a}^{3}{\prod }_{1}$ metastable state of CO measured in about 25 minutes. The transition $|v=\mathrm{1,}\,J=\mathrm{1,}-\rangle \leftarrow |v=\mathrm{0,}\,J=\mathrm{1,}+\rangle$ shows a width of 900 kHz. A Voigt fit of the data (blue line) yields an uncertainty of 3 kHz on the center frequency. Bottom: sketch of the molecular beam apparatus used for the measurement. The beam is generated by a pulsed valve operated at 10 Hz. CO molecules are skimmed, excited into the metastable state by a UV laser at 206 nm, interact with the mid IR laser, and are finally detected by resonance-enhanced multiphoton ionization. Ions are collected on a microchannel plates detector. Right: level diagram of the states involved in the triple resonance scheme.

The metastable ${a}^{3}{\Pi }_{1}$, $v=0$, $J=1$, + state is prepared from the ground state with a pulsed laser at 206 nm (1 mJ, 150 MHz bandwidth) (see Methods). The QCL drives the vibrational transition to the $v=1$, $J=1$, − state. From there, the molecules are detected by multiphoton resonance-enhanced ionization. The experiment runs at 10 Hz. The stability and robustness of this triple resonance setup allows for rapid scans yielding high signal-to-noise ratios. A 25-minutes scan yields a Doppler-broadened absorption profile with a FWHM of 900 kHz, as the one shown in Fig. 3, which is already about two orders of magnitude narrower than the best value reported in the literature (±90 MHz)^31^. We acquired 22 scans over a few days for a total measurement time of 9 hours. This set of data determines the line center of the chosen vibrational transition on the metastable excited state with an uncertainty of 3 kHz as 51399115447 kHz—an improvement of more than 4 orders of magnitude on ref.^31^. The relative uncertainty of 6 × 10^−11^ is mainly due to systematic effects to which the QCL frequency uncertainty and linewidth contributes with 2 × 10^−14^ at worst.

## Conclusions

We described a method to trace the frequency of an IR laser in the range from 1 to 10 μm with state-of-the-art metrological characteristics. Our frequency locking method needs only a little fraction of the laser power in the mid IR, leaving the rest available for spectroscopy. We use a QCL as a light source in the mid IR. The range below 3 μm, where QCLs are not available, can be covered using various commercially-available solid state lasers and very efficient non-linear crystals. Thus, our setup extends high-resolution, accurate frequency measurements to the whole molecular fingerprint region.

It has been suggested that the simultaneous monitoring of atomic and molecular transitions in various laboratories around the world can shed light on topological defect dark matter^32^. The fibre link network that is being developed on European scale, together with methods like the one presented here can contribute to such investigations. Moreover, the fibre network together with state-of-the-art mid IR photonics and innovative techniques of molecular beam manipulation brings atomic-level accuracies within reach. In particular, the 10^−16^ accuracy allowed by the fibre link can become the standard accuracy for measurements in the mid IR (~10^13^ Hz), when molecular cooling techniques will allow for 1-second interaction times.

The datasets generated during and/or analysed during the current study are available from the corresponding author on reasonable request.

## Methods

### Mid IR light source

We mix 5 W at 1064 nm from a Nd:YAG laser (InnoLight GmbH, Mephisto MOPA 55 W, ${\rm{\Delta }}\nu  \sim 1$ kHz over 100 ms) with 30 mW at 1301 nm from an extended-cavity tunable diode laser (Toptica Photonics AG, DL PRO, ${\rm{\Delta }}\nu \sim 100$ kHz) in a temperature-stabilized OP-GaP crystal (BAE System, Inc.) to obtain about 60 μW of light at 5.8 μm. The OP-GaP crystal is 24.6 mm long and has a patterned period of 24 μm in a 400 μm thick layer. We overlap the two near IR lasers with a dichroic mirror and focalize them on the crystal with a *f* = 50 mm plano-convex lens. A Germanium window separates the pump and the signal beams from the idler. The pump beam is vertically polarized (along the [001] crystallographic axis), the signal is horizontal ([110]), and they yield a horizontally polarized idler radiation. The idler power is boosted by phase locking a temperature-stabilized QCL (Alpes Laser SA) to it, which emits 10 mW at 5.8 μm. The beat note between the idler and 100 μW of the QCL radiation is detected by a thermoelectrically cooled HgCdTe photodiode with 200 μm active area (Vigo System S. A., PVI-4TE-5/MIP). The beat note has a signal-to-noise ratio of about 30 dB (on a 100 kHz bandwidth) and feeds a phase lock loop that controls the QCL current with a 300-kHz bandwidth.

### Locking chain

The carrier-envelope offset frequency *f*_0_ of a commercial frequency comb (Menlo Systems GmbH, FC1500-100-WG) is stabilized at 20 MHz using a radio-frequency reference signal delivered by a GPS-disciplined rubidium oscillator. The repetition rate frequency *f*_rep_ is phase-locked to the reference light at 1542 nm using an intra-cavity electro-optic modulator on a bandwidth of approximately 300 kHz. A short-pass dichroic mirror (cutoff wavelength of 1180 nm) splits the comb spectrum into two arms. The short-wavelength part is overlapped with the 1064 nm radiation and sent towards a diffraction gratings mounted in Littrow configuration. The diffracted comb is filtered out by an aperture and an interferometric filter that transmits the 1064 nm laser and a small part of the comb spectrum with wavelength around 1064 nm. The beat note is detected by two InGaAs photodiodes in a differential scheme (OSI Optoelectronics, FCI-InGaAs-75). A similar optical path is used to obtain the beat note between the signal laser and the long-wavelength portion of the comb spectrum. Both beat notes are detected with a minimum signal-to-noise ratio of 25 dB on a 100 kHz bandwidth.

The Nd:YAG is phase locked directly on the beat note with the comb with a bandwidth of about 700 Hz. To implement the indirect locking scheme, we first mix the pump–comb beat note with the carrier-envelop offset frequency *f*_0_ to obtain ${\bar{f}}_{{\rm{b}}:{\rm{pump}}}$. Then, a 14-bit direct digital synthesizer (Analog Devices, Inc., UG-207) creates $\frac{{N}_{{\rm{signal}}}}{{N}_{{\rm{pump}}}}{\bar{f}}_{{\rm{b}}:{\rm{pump}}}$ from ${\bar{f}}_{{\rm{b}}:{\rm{pump}}}$. Finally, we mix the signal–comb beat note with the offset frequency, obtaining ${f}_{{\rm{b}}:{\rm{signal}}}$, which is mixed with the rest yielding ${\bar{f}}_{{\rm{b}}:{\rm{signal}}}-\frac{{N}_{{\rm{signal}}}}{{N}_{{\rm{pump}}}}{\bar{f}}_{{\rm{b}}:{\rm{pump}}}$. This frequency is used as the error signal for the phase-lock loop that acts on the diode laser current with a bandwidth exceeding 200 kHz.

### Experimental Setup

A mixture of 20% CO in Kr at a backing pressure of 2 bar expands through the nozzle of a pulsed solenoid valve (Parker Hannifin Corp., modified General Valve series 99), which is kept at 140 K and operates at 10 Hz. After a 1-mm skimmer, the collimated beam has a velocity of 318 m/s. A pulsed laser at 206 nm with a fluence of 1 mJ and a bandwidth of 120 MHz transfers the CO molecules from the ground state ${X}^{1}{{\rm{\Sigma }}}^{+}$ to the upper ${\rm{\Lambda }}$-doubling component of the ${a}^{3}{\rm{\Pi }},\,v=\mathrm{0,}\,J=1$ state. Continuing their flight, the molecules interact twice with the QCL radiation that drives the vibrational transition to the $v\mathrm{=1,}\,J=\mathrm{1,}\,-$ state: a corner cube retro-reflects the QCL. Three couples of coils in Helmholtz configuration compensate the stray magnetic field in the region where the QCL interacts with the molecules.

Finally, the molecules are detected by multiphoton resonance-enhanced ionization in a 1 + 1 process at 283 nm via the intermediate ${b}^{3}{{\rm{\Sigma }}}^{+},v^{\prime} =\mathrm{1,}\,N^{\prime} =\mathrm{1,}\,-$ state. This radiation is provided by a tunable pulsed dye laser (Radiant Dyes Laser & Accessories GmbH, NarrowScan) pumped with a frequency doubled Nd:YAG laser source with 10 Hz repetition rate (InnoLas Photonics GmbH, SpitLight 1200). We used Fluorescine 27 diluted in methanol to produce radiation at about 566 nm and then we double the frequency with a BBO crystal. A few mJ suffice to saturate the ionization process. Immediately before the CO molecules are ionized, we turn on a strong electric field to accelerate the ions toward a microchannel-plate detector. Such field allows the otherwise forbidden transition between states of the same parity.

The 206 nm and 5.8 μm beams are parallel, while the 283-nm beam is perpendicular to them. We use a micrometric translation stage to finely change the position of the ionization laser along the direction parallel to the mid IR laser.

### Uncertainty on the spectroscopic measurements

A corner cube retro-reflects the QCL beam. The two anti-parallel laser beams induce Doppler shifts on the transition that are equal in magnitude but opposite in sign. Thus, when the molecular beam and the mid-IR laser beam are not perpendicular, we observe a symmetric splitting of the absorption line. We record the magnitude of the splitting in dependence on the angle between the laser beam and the molecular beam, and, finally, we settle at the position corresponding to the minimal splitting. The remaining systematic uncertainty is due to imperfect parallelity of the counter-propagating mid-IR beams. This has been measured and it is better than 10^−4^ rad, which corresponds to a final uncertainty on the transition frequency of 2.6 kHz.

We estimate the Stark and second-order Zeeman shifts due to stray fields to be lower than 10 Hz. Since we prepare and detect the molecules with focused ns lasers at precisely-known times and positions, we know the speed of the molecules, 318.5 ± 2 m/s. Thus, we calculate the second-order Doppler shift, +29.4 ± 0.4 Hz, and we subtract it from the measured frequency. The remaining systematic uncertainty is due to the Cs fountain standard accuracy^33^ and to fibre link phase slips^23^, both smaller than 100 mHz. Therefore, the *total systematic uncertainty* is estimated as 2.6 kHz.

The Zeeman shift of the ${\rm{\Delta }}M=\pm 1$ is of the order of 500 kHz/Gauss, whereas the ${\rm{\Delta }}M=0$ are shifted by about 10 kHz/Gauss. By canceling the Zeeman shift on the ${\rm{\Delta }}M=\pm 1$ transitions, we estimate that the residual Zeeman shift on the ${\rm{\Delta }}M=0$ transition is smaller than 1 kHz. We repeated the canceling procedure several times between the scans and we found no variation of the compensating magnetic field. This contributes to the statistical uncertainty, together with the uncertainty in the alignment of the beams, whose procedure is described above, and with the fluctuations in the population of metastable CO molecules. The distribution of the experimental measurements has a Gaussian shape with a standard deviation of 7.9 kHz. Our set of 22 scans yields a *total statistical uncertainty* of 1.7 kHz.

## References

[1] Nicholson, T. L. *et al*. Systematic evaluation of an atomic clock at 2 × 10^–18^ total uncertainty. *Nature Comm.* **6**, 6896 (2015). 10.1038/ncomms7896 PMC4411304 25898253

[2] Baron J (2013). Order of magnitude smaller limit on the electric dipole moment of the electron. Science.

[3] Shelkovnikov A, Butcher RJ, Chardonnet C, Amy-Klein A (2008). Stability of the proton-to-electron mass ratio. Phys. Rev. Lett..

[4] Truppe S (2013). A search for varying fundamental constants using hertz-level frequency measurements of cold CH molecules. Nature Comm..

[5] Darquié B (2010). Progress toward the first observation of parity violation in chiral molecules by high-resolution laser spectroscopy. Chirality.

[6] Salumbides EJ, Dickenson GD, Ivanov TI, Ubachs W (2011). QED effects in molecules: Test on rotational quantum states of H_2_. Phys. Rev. Lett..

[7] Salumbides EJ (2013). Bounds on fifth forces from precision measurements on molecules. Phys. Rev. D.

[8] Borri S, Santambrogio G (2016). Laser spectroscopy of cold molecules. Advances in Physics: X.

[9] Hummon MT (2013). 2d magneto-optical trapping of diatomic molecules. Phys. Rev. Lett..

[10] Zhelyazkova V (2014). Laser cooling and slowing of CaF molecules. Phys. Rev. A.

[11] Barry JF, McCarron DJ, Norrgard EB, Steinecker MH, DeMille D (2014). Magneto-optical trapping of a diatomic molecule. Nature.

[12] Prehn A, Ibrügger M, Glöckner R, Rempe G, Zeppenfeld M (2016). Optoelectrical cooling of polar molecules to submillikelvin temperatures. Phys. Rev. Lett..

[13] Cheng C (2016). Molecular fountain. Phys. Rev. Lett..

[14] Truppe S (2017). Molecules cooled below the Doppler limit. arXiv.

[15] Ricciardi I (2015). Sub-kilohertz linewidth narrowing of a mid-infrared optical parametric oscillator idler frequency by direct cavity stabilization. Opt. Lett..

[16] Vitiello MS, Scalari G, Williams B, De Natale P (2015). Quantum cascade lasers: 20 years of challenges. Opt. Express.

[17] Bartalini S (2010). Observing the intrinsic linewidth of a quantum-cascade laser: Beyond the Schawlow-Townes limit. Phys. Rev. Lett..

[18] Borri S (2011). Frequency-noise dynamics of mid-infrared quantum cascade lasers. IEEE J. Quant. Elec..

[19] Cappelli F (2012). Subkilohertz linewidth room-temperature mid-infrared quantum cascade laser using a molecular sub-Doppler reference. Opt. Lett..

[20] Hansen, M. G., Magoulakis, E., Chen, Q.-F., Ernsting, I. & Schiller, S. Quantum cascade laser-based mid-IR frequency metrology system with ultra-narrow linewidth and 1 × 10^−13^-level frequency instability. *Opt. Lett.* **40**, 2289 (2015). 10.1364/OL.40.002289 26393721

[21] Argence B (2015). Quantum cascade laser frequency stabilization at the sub-Hz level. Nature Phot..

[22] Galli I (2013). Comb-assisted subkilohertz linewidth quantum cascade laser for high-precision mid-infrared spectroscopy. Appl. Phys. Lett..

[23] Clivati C (2016). Measuring absolute frequencies beyond the GPS limit via long-haul optical frequency dissemination. Opt. Express.

[24] Calonico D (2014). High-accuracy coherent optical frequency transfer over a doubled 642-km fiber link. Appl. Phys. B.

[25] Insero G (2016). Difference frequency generation in the mid-infrared with orientation-patterned gallium phosphide crystals. Opt. Lett..

[26] Pomeranz LA (2015). 1-*μ*m-pumped OPO based on orientation-patterned GaP. Proc. of SPIE.

[27] Petrov V (2015). Frequency down-conversion of solid-state laser sources to the mid-infrared spectral range using non-oxide nonlinear crystals. Prog. Quantum Electron..

[28] Williams PA, Swann WC, Newbury NR (2008). High-stability transfer of an optical frequency over long fiber-optic links. J. Opt. Soc. Am. B.

[29] Telle HR, Lipphardt B, Stenger J (2002). Kerr-lens, mode-locked lasers as transfer oscillators for optical frequency measurements. Appl. Phys. B.

[30] Hansen MG (2013). Robust, frequency-stable and accurate mid-IR laser spectrometer based on frequency comb metrology of quantum cascade lasers up-converted in orientation-patterned GaAs. Opt. Express.

[31] Davies, P. B. & Martin, P. A. Diode-laser spectroscopy of a^3^Π CO. *Mol. Phys.* **70**, 89 (1990).

[32] Stadnik YV, Flambaum VV (2014). New atomic probes for dark matter detection: Axions, axion-like particles and topological defects. Mod. Phys. Lett. A.

[33] Levi F (2014). Accuracy evaluation of ITCsF2: a nitrogen cooled caesium fountain. Metrologia.

